# Global multi-environment resistance QTL for foliar late blight resistance in tetraploid potato with tropical adaptation

**DOI:** 10.1093/g3journal/jkab251

**Published:** 2021-08-05

**Authors:** Hannele Lindqvist-Kreuze, Bert De Boeck, Paula Unger, Dorcus Gemenet, Xianping Li, Zhechao Pan, Qinjun Sui, Junhong Qin, Gebremedhin Woldegjorgis, Kassaye Negash, Ibrahim Seid, Betaw Hirut, Manuel Gastelo, Jose De Vega, Merideth Bonierbale

**Affiliations:** 1 International Potato Center, CIP, Lima 15024, Peru; 2 CIP Kenya, c/o ILRI, P.O. Box 30709, Nairobi, Kenya; 3 ndustrial Crops Research Institute, Yunnan Academy of Agricultural Sciences (YAAS), 2238 Beijing Road, Kunming, Yunnan 650205, P. R. China; 4 CIP China, Beijing 100081, China; 5 Ethiopian Institute of Agricultural Research, (EIAR), Holetta Agricultural research Center. P.O. Box 31, West Showa Zone, Oromia Region, Ethiopia; 6 CIP Ethiopia, c/o ILRI Ethiopia P.O. Box 5689, Addis Ababa, Ethiopia; 7 Earlham Institute (EI), Norwich Research Park, Norwich NR4 7UZ, UK

**Keywords:** GWAS, polyploidy, breeding, genotyping by sequencing (GBS), *Phytophthora infestans*, GBLUP

## Abstract

The identification of environmentally stable and globally predictable resistance to potato late blight is challenged by the clonal and polyploid nature of the crop and the rapid evolution of the pathogen. A diversity panel of tetraploid potato germplasm bred for multiple resistance and quality traits was genotyped by genotyping by sequencing (GBS) and evaluated for late blight resistance in three countries where the International Potato Center (CIP) has established breeding work. Health-indexed, *in vitro* plants of 380 clones and varieties were distributed from CIP headquarters and tuber seed was produced centrally in Peru, China, and Ethiopia. Phenotypes were recorded following field exposure to local isolates of *Phytophthora infestans*. QTL explaining resistance in four experiments conducted across the three countries were identified in chromosome IX, and environment-specific QTL were found in chromosomes III, V, and X. Different genetic models were evaluated for prediction ability to identify best performing germplasm in each and all environments. The best prediction ability (0.868) was identified with the genomic best linear unbiased predictors (GBLUPs) when using the diploid marker data and QTL-linked markers as fixed effects. Genotypes with high levels of resistance in all environments were identified from the B3, LBHT, and B3-LTVR populations. The results show that many of the advanced clones bred in Peru for high levels of late blight resistance maintain their resistance in Ethiopia and China, suggesting that the centralized selection strategy has been largely successful.

## Introduction 

Potato *genetic* resources comprise a polyploid series consisting of tremendously diverse germplasm of wild relatives and cultivated landraces (Ovchinnikova *et al.* 2011; Spooner *et al.* 2014). However, most commercially cultivated potato varieties are tetraploid (2*n* = 4x = 48) with the genome consisting mostly of *Solanum tuberosum* Group Tuberosum with some introgressions from a few wild species and cultivated landraces (Bradshaw *et al.* 2006; reviewed by Bethke *et al.* 2017; reviewed by Gaiero *et al.* 2018). Tetraploid potato is a highly heterozygous, outcrossing autopolyploid crop, which complicates genetic analysis. Most early genetic mapping studies utilized bi-parental populations at the simpler, diploid level (2*n* = 2x = 24) and several disease resistance loci have been identified this way (reviewed by Gebhardt and Valkonen 2001; Tiwari *et al.* 2013). However, this approach does not permit the assessment of large gene pools or multi-allelic interactions that influence traits in polyploids. Significant progress has recently been made in the development of algorithms and software for genotype calling, linkage, and QTL analysis in polyploid species.

Some single nucleotide polymorphism (SNP) arrays have been developed for potato: 8 K SolCAP (Hamilton *et al.* 2011; Felcher *et al.* 2012) and the 20 K SolSTW arrays (Uitdewilligen *et al.* 2013; Vos *et al.* 2015). These were developed using North American and European potato germplasm, respectively, and are consequently not the best options for genotyping CIP germplasm, which contains more introgressions from the South American gene pool. According to our previous experience, less than 50% of the SNPs on the 8 K SolCAP array were informative in a test sample of CIP germplasm (Lindqvist-Kreuze *et al.* 2014). Genotyping by sequencing (GBS) has been applied to tetraploid potato (Uitdewilligen *et al.* 2013; Sverrisdóttir *et al.* 2017) and variant calling from short-read sequencing data considering allele dosage is now possible using several different tools, such as GATK, Freebayes, or SAMtools, to name a few (Clevenger *et al.* 2015). However, reliable dosage calling in the heterozygous individuals depends on the read depth in the SNP loci. It was recently demonstrated in autopolyploid forage grass *Urochloa* spp. that a read depth of 61 was adequate to reliably call allele dosage, while only 17 reads were needed to reliably classify simplex tetraploids as heterozygous with 95% accuracy (Matias *et al.* 2019). The identification of QTL in autopolyploids is facilitated by new tools, such as GWASpoly, that consider allele dosage effects (Rosyara *et al.* 2016). Together, these advances make genomic analysis of tetraploid potato more informative and applicable to evolutionary and breeding studies.

The goal of CIP’s potato breeding program is to develop resilient, high-yielding, nutritious, and early-maturing varieties for smallholder farming systems in the developing world. We are targeting farming systems that must function with minimum input of pesticides; therefore, a high level of disease resistance is an indispensable trait. To this end, CIP’s potato breeding program has developed breeding populations with high levels of resistance to late blight caused by the oomycete *Phytophtora infestans*, and resistance to Potato Virus Y (PVY), Potato Virus X (PVX), and Potato Leaf Roll Virus (PLRV). Previous studies have identified genomic regions in CIP-bred germplasm to explain resistance to late blight focusing on phenotypic data collected from field trials in Peru or using local pathogen strains in greenhouse conditions (Li *et al.* 2010; Lindqvist-Kreuze *et al.* 2014; Jiang *et al.* 2018). Information on the late blight resistance of CIP-bred materials has been published sporadically in target regions where they have been distributed (Li *et al.* 2018; Sun et al. 2010; Muhinyuza *et al.* 2015; Hirut *et al.* 2017b); however, to our knowledge, no genetic analysis has been published that identifies QTL for resistance in CIP germplasm tested in environments outside Peru.

A major objective of this research was to evaluate the potential of genomic-based selection methods to support a global breeding strategy using late blight resistance as a case study. To systematically evaluate CIP’s breeding materials in diverse environments, we established a trait observation network (TON) of collaborators and assembled a diversity panel that consists of representative advanced clones including elite materials from each of CIP’s breeding populations. This so-called TON panel was then distributed from Peru to China and Ethiopia, where it was included in a series of trait evaluation experiments by national research institutions and CIP. The specific aims of this study were (i) to identify QTL underlying late blight resistance in and across environments; and (ii) to test prediction models to support the global deployment and use of promising resistance sources in local breeding and variety development programs.

We report the genotyping, estimation of linkage disequilibrium, and population structure of the TON panel and identify QTL for late blight resistance via genome-wide association (GWA). In addition, we present a case for genomics-assisted breeding for foliar late blight resistance and show how the use of genomics and pedigree information can be used to select “best bet” clones for breeding and variety development in diverse target environments.

## Materials and methods

### Germplasm

The TON panel consisted of 380 genotypes representing seven CIP breeding populations and a group of varieties with variable origins (Table 1). “Population A” was developed at CIP between 1980 and 1990 with emphasis on late blight resistance. Sources of late blight resistance were improved materials with *S. demissum*-derived resistance from breeding programs around the world, including native Andean cultivars (*S. tuberosum* groups Andigena, Phureja, and Stenotomum) and wild species (*S. acaule* and *S. bulbocastanum*). “Population B3” genotypes were derived from “Population A” with emphasis on increasing frequencies and levels of quantitative resistance to late blight. The “B1 population” is derived from the *S. tuberosum* group, Andigena. The “LTVR population” is characterized primarily for its resistance to the most important virus diseases (PVY, PVX, and PLRV), short crop duration, and adaptability to warm environments. The “LB-HT” population combines late blight resistance from the “B3 population” and heat tolerance from North American and European-bred varieties and the LTVR population. The “B3-LTVR” population contains hybrid genotypes originating from crosses between “B3” and “LTVR populations.” The “pre-Bred” population has genotypes with late blight resistance introduced from wild *Solanum* species into the tetraploid background of “B3” or “LTVR.” The varieties group consists of several potato varieties or key breeding lines: “Desiree, “Atlantic,” “Spunta,” “Granola,” “Yungay,” “Tomasa Condemayta,” “DTO-33,” “Kufri Yoti,” and “Chucmarina.” The CIP numbers and the parentage of the 380 genotypes are given in Supplementary Table S1.

**Table 1:** CIP potato programs breeding populations utilized in the study, the main breeding objective of the populations, and the number of genotypes derived from each population and subpopulation

Breeding population (subpopulation)	Genotypes evaluated	Years of crosses made	Main breeding objective
A	13	1974, 1977, 1980, 1981, 1984, 1987, 1991	Late blight resistance, adaptation to highlands
B1	11	1999	
B3 (B3C0, B3C1, B3C2)	100 (2, 51, 47)	1987, 1989, 1991, 1992, 1993, 1995, 1996	
LB-HT	37	1998	Late blight resistance, heat tolerance, adaptation to mid-elevation
B3-LTVR (intermediate-LT-LB, XY-LB)	25 (12, 13)	1996, 2001	Hybrid population combining late blight resistance, heat tolerance, virus resistance
LTVR (LTVR, BW)	186 (171, 15)	1979, 1980, 1985, 1988, 1989–2000, 2002–2004, 2009	Virus resistance, heat tolerance, drought tolerance, salinity tolerance, adaptation to lowlands, bacterial wilt resistance
PREBRED	2	1994	Late blight resistance
VARIETY	6	NA	Varied
Grand total	380		

### Environments

The field sites in Ethiopia and China are within important potato production areas. In the Peruvian field site, potato is not the main crop, but late blight is endemic with high pressure whenever the crop is grown (Table 2). The late blight pathogen populations have been described in each location. In Peru and Ethiopia, only the A1 mating type has been identified and different clonal lineages are present, frequently containing virulence to most of the known *S. demissum R* genes (Lindqvist-Kreuze *et al.* 2020; Mihretu et al. 2020). Meanwhile, the A2 mating type has been found to dominate in southern China (Li *et al*. 2013). All locations are in tropical and subtropical regions with latitudes ranging between 9°N and nearly 25°S.

**Table 2:** Geographical location, year of implementation, and detailed description of the design of the field trials conducted to obtain estimates of late blight resistance of the potato genotypes in China, Ethiopia, and Peru

Country	Location	Year	Number of genotypes evaluated (statistical design)	Checks and shared varieties	H2 rAUDPC (Cullis et al. 2016)
Peru	Pasco, Oxapampa 10.5853°S, 75.4053°W	2014	240 (resolvable incomplete block design; 3 replicates)	Chucmarina Unica Tomasa Desiree Spunta, Granola, Atlantic	0.99
China	Yunnan, Kunming 24.8801°N, 102.8329°E	2015	306 (RCBD; 3 replicates)	Chucmarina C-88 Unica Desiree, Kexin, Yunshu, Zhongsu, Tomasa	0.95
		2016	336 (RCBD; 3 replicates)		0.96
Ethiopia	Oromia, Holetta 9.0633°N, 38.4902°E	2017	60 (RCBD; 3 replicates)	Belete Gudene Jalene Unica	0.98
		2016	128 (CRD; 1.63*a* replicates)	Gudene Belete Jalene Unica Tomasa	0.96

The estimate of the heritability of resistance based on the relative Area Under the Disease Progress Curve (rAUDPC) according to Cullis *et al.* (2016) for each trial is shown.

a The trial design does not have complete replicates. Therefore, the theoretical replication number is calculated as the harmonic mean of the number of replications across all genotypes.

RCBD, randomized complete block design; CRD, completely randomized design.

### Field trials

Standard protocols at CIP were followed for planning and conducting the field trials (Forbes *et al.* 2014). The statistical designs in each trial are shown in Table 2. Uniform tuber seed was produced centrally in each country following the introduction of *in vitro* plants or mini-tubers from CIP facilities in Peru or Kenya.

Late blight resistance was evaluated under endemic disease pressure. The disease level in the plots was recorded as “percent leaf area infected” at typically 7-day intervals until susceptible controls reached 100% infection. These values were used to calculate the area under the disease progress curve (AUDPC) and relative AUDPC (rAUDPC). AUDPC is expressed in infection percentage times days (Forbes *et al.* 2014), while the rAUDPC was calculated by dividing the AUDPC by the “maximum potential AUDPC,” which is the AUDPC a genotype would have if it had 100% infection at all readings.

### Genotyping, variant calling, and filtering for association analysis

In total, 380 potato clones were genotyped. Library construction and GBS were outsourced to the Genomics Facility at Cornell University in 2015. The DNA was digested with *EcoT*221 restriction enzyme and the libraries were 48x multiplexed for sequencing. The diploid calling was performed using the Tassel pipeline (Bradbury *et al.* 2007). The resulting Variant Call Format (VCF) file was processed with Bcftools (https://samtools.github.io/bcftools/) to filter the variants for a minimum read depth (RD) of 17, a minimum genotype quality (GQ) of 30, and a minor allele frequency (MAF) of 0.03. The SNPs that did not pass these criteria were changed to missing calls. Only the SNP sites that contained less than 30% missing data were selected.

For polyploid calling, the raw FASTQ files were processed with Stacks (Catchen *et al.* 2013) to remove the barcodes and TrimGalore was used (https://github.com/FelixKrueger/TrimGalore) to trim the ends of the reads. The reads were aligned to the reference genome version *S. tuberosum_448_v4.03* (Sharma *et al.* 2013) using BWA (Li and Durbin 2009) and the resulting SAM files were converted to BAM files using Samtools (Li *et al.* 2009). The variants were called using the GATK HaplotypeCaller option (Poplin et al. 2018), disabling the duplicate read filter (as recommended for GBS data), and joint genotyped using the -ERC GVCF mode. From the VCF files, SNP calls were filtered using Bcftools for a minimum RD of 61, a minimum GQ of 30, and a MAF of 0.03. The samples that did not surpass these criteria were changed to missing calls. Only the SNP sites that contained less than 30% missing data were included in the analysis.

### Analysis of the population substructuring

Population structure was analyzed on a subset of 7597 diploid markers with no missing data. First, K-means clustering over *K*-values from 1 to 20 was repeated 100 times to identify potential clusters. The values K2-9 were further selected for discriminant analysis of principal components (DAPC) and for each K, the potato genotypes (clones) were assigned in the K groups based on the highest probability. To estimate the components of covariance among and within the clusters Analysis of molecular variance (AMOVA) was conducted using a farthest-neighbor algorithm. K-means clustering and AMOVA were conducted using Poppr v 2.9.0 (Kamvar *et al.* 2014).

### Linkage disequilibrium decay

Correlations between all pairs of markers were calculated using Pearson’s correlation coefficient (*r*^2^) based on the SNP dosage scores (0–4) and plotted against the physical distance of the marker pairs. A spline was fitted on the 90th percentile using quantile regression (Koenker 2017) and the estimators for the LD decay were obtained from the spline at *r*^2^ of 0.1. In addition, another estimate for LD decay was obtained from the fitted spline: the distance at which half of the short-range LD had decayed based on the 90^th^ percentile, LD_½,90_ as described by Vos *et al.* (2017).

### Genome-wide association

Marker trait associations were modeled for all trials independently with the GWASpoly package using both diploid and tetraploid marker sets (Rosyara *et al.* 2016). The BLUE values were used as phenotypic values for each experiment (Supplementary Table S2). For the tetraploid dataset, general, additive, simplex dominant (1-dom), and duplex dominant (2-dom) models were used while for the diploid dataset, diplo-general, diplo-additive, and the simplex dominant (1-dom) models were used. The parameters used for the GWAS modeling function (*GWASpoly*) in R were the following: no additional fixed effects; four principal components included as covariates to account for the population substructuring; a minimum MAF of 0.03; a maximum genotype frequency (after applying dominance relations) of 0.95; and P3D approximation. To detect statistical significance, the Bonferroni correction method was used, ensuring the genome-wide type I error would not be greater than 0.05. Manhattan plots were generated to display significant SNP in the different genetic models. In addition, Quantile–Quantile (Q–Q) plots of the observed *vs* expected –log10(p) values were used to evaluate that the population structure had been adequately controlled.

The genomic positions of the QTL, known genes, and the SNPs associated with plausible QTL for pathogen resistance in relation to other loci were determined using the genome browser available at http://solanaceae.plantbiology.msu.edu/ and *S.* *tuberosum* Group Phureja DM1-3 516R44 v4.03 pseudomolecules. To approximate the physical location of markers for pathogen resistance present in literature, the position in cM was obtained from the GABI Primary Database (https://www.gabipd.org/projects/Pomamo/) and then translated to an approximate physical position in Mbp using the information provided in Sharma *et al.* (2013) that integrates the potato genome and physical and genetic maps.

### Statistical analysis of phenotypic data

From the weekly observations of the disease incidence in the plots, the AUDPC was calculated and then rescaled to the relative AUDPC (rAUDPC) to facilitate comparison among the different environments. Because the experimental design types varied across environments, the raw data were analyzed using a stage-wise approach as elaborated in Piepho *et al.* (2012) and van Eeuwijk *et al.* (2016). In the first stage, genotypic best linear unbiased estimators (BLUEs) of rAUDPC for each environment separately were obtained by fitting a mixed model that considered the experimental design used in the specific environment (results presented in Supplementary Table S2). Single trial broad-sense heritabilities (H^2^) were estimated for rAUDPC using the methodology proposed by Cullis *et al.* (2006) (Table 2).

In the second stage, the genotype-by-environment table of BLUEs and their standard errors were used as starting point for the multi-environment trial (MET) analysis. The BLUEs were weighted according to Method 2, as described in Möhring and Piepho (2009), to fit a mixed model that considers possible genotype-by-environment interaction (GEI). In the presence of GEI, the more realistic models often allow for heterogeneity of genetic variances and covariances across environments (Malosetti *et al.* 2013). The best-fitting model was chosen using Akaike’s information criterion (AIC): µ_*ij*_ = µ + *G_ij_* + *E_j_* + ɛ_*ij*_ where µ is the overall mean (intercept), *E_j_* the fixed effect of the j^th^ environment (j=1, …,5), *G_ij_* the random environment-specific genetic effect of the i^th^ genotype in environment j and ɛ_*ij*_ ∼ N(0, 1) standard normally distributed and independent random error effects (for all i=1, …,I and j=1, …,5). The underlined terms in the model are random effects and all other terms are fixed effects. Note that observations µ_*ij*_ are the reweighted BLUEs. In contrast to the residual errors, the random genetic effects are not necessarily independent for all i and j. The 5-dimensional random genetic effect ***G****_i_* = (*G_i1_*,…, *G_i5_*)^T^ of the i^th^ genotype follows a multivariate normal distribution ***G***_*i*_ ∼ N(0, $\sum_{E}$) with variance-covariance matrix $\sum_{E}$ allowing for flexible GEI modelling. The variance-covariance matrix $\sum_{E}$ of the random genetic effect ***G***_*i*_ was parametrized using the second-order factor analytic model that accommodates heterogeneity of genetic variances and genetic covariances across environments in a parsimonious manner (Piepho 1998). The concatenated random effects vector ***G*** = (***G***_*1*_,…,***G***_*I*_)^T^ was assumed to follow a multivariate normal distribution with mean **0** and variance-covariance matrix ∑ where $\sum =\;\sum_{G}⊗\sum_{E}$. The symbol ⊗ denotes the Kronecker product and $\sum_{G}$ denotes the kinship matrix defining the genetic relationships between the different genotypes. The matrix $\sum_{E}$ was estimated when fitting the model, but the I x I matrix $\sum_{G}$ had to be stated as prior knowledge before fitting the model. Choosing a different approach to determine this matrix $\sum_{G}$, therefore, led to different kinds of final models. The considered options are described below.

In a first simplified approach, it was assumed that $\sum_{G}=I_{I}$, where $I_{I}$ is the I - dimensional identity matrix. The corresponding model therefore assumes that there are no genetic correlations between genotypes, but the final variance-covariance matrix ∑ of the random genetic effect ***G*** allows for heterogeneity of genetic variances and covariances across environments through $\sum_{E}$. The genotypic best linear unbiased predictors (BLUPs), predicting rAUDPC per genotype and possibly per environment, were obtained from the selected mixed model that describes the genetic effect and the GEI in terms of heterogeneity of variances and covariances across environments.

Alternative—and more realistic—approaches recognize that genetic relationships between the genotypes exist. In the following models, genetic relationships were incorporated, first by using genetic theory (pedigree-based relationship or kinship matrix) and then by using the available molecular information (molecular-based relationship or kinship matrix). In the standard approach described above, the random genetic effects ***G***_*i*_ are assumed independent for i=1, …,I. It is, however, more realistic to consider a variance-covariance structure for the genetic effects that integrates the expected or observed relationships between genotypes. In the first case, the relationship matrix $\sum_{G}$ was calculated using pedigree of up to four generations deep that was not complete for all genotypes. The resulting mixed model is called the PBLUP model. Four GBLUP models were fitted calculating the relationship matrix $\sum_{G}$ based on the same 2x and 4x marker sets as used in the GWA analysis. For both ploidy levels, a GBLUP model was considered without including the diagnostic SNP markers as fixed effects in the model, and a separate GBLUP model was considered by including the diagnostic SNP markers listed in Tables 4 and 5 as fixed effects in the model and testing for the significance of these QTL and QTL-by-environment (QTLxE) interaction effects. Using the tetraploid SNP marker data, this approach led to a final GBLUP model with fixed effects for the dosage of 0_36073482, 3_3319097 and 9_60067335 SNP markers. Using the diploid SNP marker data, this approach led to a final GBLUP model with fixed effects for the dosage of 0_36073482, 5_5572873, 9_59967523 and 9_60067335 SNP markers. The pedigree BLUPs (PBLUPs) and the genomic BLUPs (GBLUPs), predicting rAUDPC per genotype and possibly per environment, were obtained from the selected mixed model that describes the genetic effect and the GEI in terms of heterogeneity of variances and covariances across environments, but also accounts for the correlations between genotypes within environments.

**Table 3:** Analysis of molecular variance (AMOVA) to test for population differentiation among the potato genotypes based on K-means clustering of *n* = 2–9

Variance components	*K* = 2	*K* = 3	*K* = 4	*K* = 5	*K* = 6	*K* = 7	*K* = 8	*K* = 9
σ^2^_p_	0.00231	0.003557	0.10983	0.10818	0.10580	0.06733	0.06439	0.05695
σ^2^_g(p)_	0.04507	0.04385	0.01477	0.00958	0.00721	0.00723	0.00725	0.00728
σ^2^_p_/(σ^2^_p +_ σ^2^_g(p) +_ σ^2^_g_)	0.04619	0.07110	0.86318	0.89853	0.91492	0.87225	0.86982	0.84989

**Table 4:** SNP markers that were significantly associated with the late blight resistance phenotype in the field trials in China (Kun2015, Kun2016), Ethiopia (Hol2016), and Peru (Oxa2014)

Marker (chromosome followed by position)	Ref	Alt	Trial	Model	Threshold	Score	Effect
0_36073482	G	A	Oxa2014	General	4.71	9.13	NA
				Additive	4.78	9.13	−0.19
				1-dom-alt	4.71	9.13	−0.19
			Kun2015	General	4.74	19.23	NA
				Additive	4.78	19.23	−0.29
				1-dom-alt	4.71	19.23	−0.29
			Kun2016	General	4.76	22.22	NA
				Additive	4.78	22.22	−0.27
				1-dom-alt	4.71	22.22	−0.27
3_3319097	A	T	Hol2017	General	4.49	5.83	NA
9_58779951	G	A	Oxa2014	Additive	4.78	4.95	−0.11
				1-dom-alt	4.71	5.62	−0.13
			Kun2015	1-dom-alt	4.71	5.53	−0.14
			Kun2016	1-dom-alt	4.71	5.01	−0.12
9_59967523	A	T	Kun2015	General	4.74	7.5	NA
				Additive	4.78	6.92	−0.14
				1-dom-alt	4.71	8.39	−0.18
			Kun2016	General	4.76	8.59	NA
				Additive	4.78	9.18	−0.14
				1-dom-alt	4.71	9.15	−0.16
9_60067335	A	G	Oxa2014	General	4.71	11.94	NA
				Additive	4.78	12.54	−0.21
				1-dom-alt	4.71	12.94	−0.21
			Kun2015	General	4.74	18.71	NA
				Additive	4.78	18.29	−0.26
				1-dom-alt	4.71	19.61	−0.27
			Kun2016	General	4.76	19.13	NA
				Additive	4.78	19.94	−0.24
				1-dom-alt	4.71	20.18	−0.25

The associations were modeled using tetraploid SNP with GWASpoly software and general, additive, and 1-dominance models.

A fivefold cross-validation was performed to compare the different prediction models for genetic rAUDPC predictions per environment. For this validation procedure, the set of available adjusted phenotypic values (the environment-specific genetic BLUEs of all five environments) was randomly subdivided into five disjoint subsets. The environment-specific genetic rAUDPC values of each of the five subsets were predicted while the other four subsets were used as training data set to fit the prediction model. This cross-validation procedure was repeated 100 times to obtain an empirical sampling distribution of the prediction ability (*i.e.*, the Pearson correlation between the adjusted phenotypic values and the validation predictions of the genetic values). The mean and standard deviation of this empirical distribution were calculated to characterize the expected value and variability of the prediction ability, respectively.

All calculations, fitting the mixed models and calculating BLUEs and BLUPs, were done by using the ASREML-R package in R.

## Results and discussion

### SNP markers

The tetraploid dataset included a total of 305,345 SNPs after GATK variant calling, while the diploidized dataset after Tassel pipeline had an SNP count of 312,727. After applying filtering parameters, including the read depth thresholds recommended based on different ploidy levels (Matias *et al.* 2019), the number of SNPs were reduced to 3239 tetraploid SNPs and 55,748 diploid SNPs.

The number of SNP obtained through GBS depends on the complexity of the genome, restriction enzyme(s) used, and the sequencing depth. Although GBS methodology was initially developed using a single restriction enzyme (Elshire *et al.* 2011), it was soon followed by a method that uses two enzymes (Poland *et al.* 2012). In tetraploid potato, *Ape*KI or *Msp*I/*Pst*I combination has been reported (Sverrisdóttir *et al.* 2017; Bastien *et al.* 2018). It was shown that by using a single enzyme, more markers can be obtained, albeit with lower read coverage per marker as compared to the two-enzyme combo (Bastien *et al.* 2018). Thus, the single enzyme choice in our study may have contributed to low read coverage and thereby loss of markers after the subsequent filtering steps.

### Population structure

**Figure 1:**
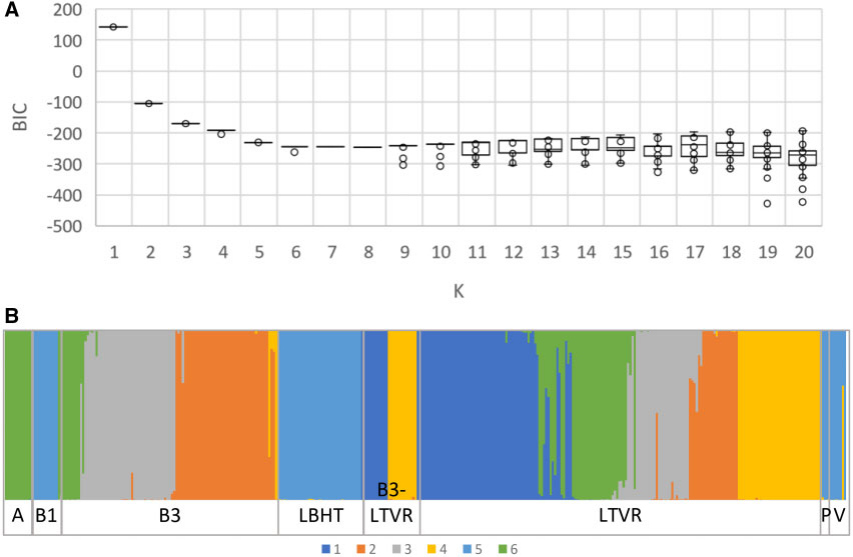
Population substructuring of the potato genotypes based on K-means clustering and discriminant analysis of principal components (DAPC). (A) K-means clustering over 20 K values. (B) The posterior probabilities of the group assignments of each potato genotype for ***K* = **6. The potato genotypes were derived from pre-defined CIP potato breeding populations [A, B1, B3, LBHT, B3_LTVR, PREBRED (=P)] and from a group of old varieties (V). See Table 1 for details.

Population structure that is not considered in GWA may result in the discovery of false-positive associations. Previous studies in tetraploid potato report either weak (D’hoop *et al.* 2010; Uitdewilligen *et al.* 2013; Vos *et al.* 2017) or absence of population structure (Li *et al.* 2010; Fischer *et al.* 2013, Stich *et al.* 2013). Based on K-means clustering and the lowest BIC value, the optimal number of subgroups in the CIP TON panel was ***K* = **6 (Figure 1A). The result was corroborated by AMOVA since with ***K* = **6, 91.5% of the variance was found between the groups (Table 3). However, these six subgroups do not strictly follow the pre-assigned breeding populations listed in Table 1 (Figure 1B). For instance, the LTVR breeding population splits into five groups, while the B3 population consists of four groups, which are also found in the LTVR population. As any long-term breeding program, CIP's potato breeding efforts have addressed a wide and dynamic range of improvement goals throughout its approximately 40-year history. The germplasm characterized in this paper is a cross-section of advanced clones from crosses made between 1974 and 2009. During this time priorities have shifted several times and new trait sources have been incorporated that can be expected to influence the composition of breeding populations. The main drivers of the trait prioritization include growing awareness and increased understanding of (i) pathogen dynamics and host-pathogen interactions driving the need for new sources of resistance to late blight; coincident with donor support for pre-breeding; (ii) global warming trends and climate extremes requiring greater tolerance for heat and drought, and (iii) political influences that change development agendas to new world regions and thus growing conditions.

The substructure encountered was included as a co-factor in the GWA to avoid false-positive associations. This was done directly in the GWASPoly package by incorporating the first four principal components in the mixed model.

### Linkage disequilibrium

**Figure 2:**
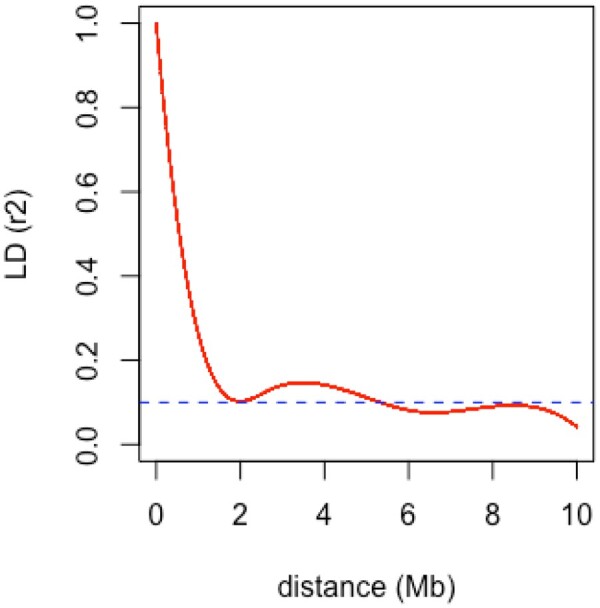
Linkage disequilibrium (LD) decay estimated in the potato genotypes. Pearson correlation coefficient (*r*^2^) was plotted against the physical map distance (Mb) between pairs of SNP. The red line is a regression spline fitted on the 90th percentile and the blue dotted line indicates the *r*^2^ value of 0.1.

The extent to which LD persists when the physical distance between the SNP markers increases is used to measure the rate of LD decay. The rate of LD decay is directly linked to the feasibility to assess marker-trait associations, since crops with low LD decay rates require lower marker density as compared to those with a faster decay. To be able to compare the LD in our potato germplasm with other recently published studies in tetraploid potato (Vos *et al.* 2017; Sharma *et al.* 2018) we estimated the LD decay on a short distance of 10 Mb. By plotting the pairwise r^2^ values with the physical distance of the markers and fitting a regression spline on the 90th percentile of the observations, we obtained an estimate for the LD decay with the *r*^2^ value of 0.1 at 2 Mb (Figure 2). This physical distance is equivalent to a genetic distance of 5cM, similar to that observed by D’hoop *et al.* (2010) in a collection of tetraploid potato cultivars representative of the cultivated gene pool in Europe and North America. As suggested by Vos *et al.* (2017), more accurate estimates may be obtained when using the LD-decay estimate value for *r*^2^_1/2max, 90_. This estimate in CIP germplasm was 0.55 Mb, which corroborates values reported in recent European potato varieties ( 0.6 Mb in Vos *et al.* 2017; 0.91 Mb in Sharma *et al.* 2018 ). The average r^2^ for the short distance in our dataset was 0.091, which is a bit lower than the average *r*^2^ (0.19–0.22) reported for European varieties (Vos *et al.* 2017), indicating that there were probably more founder haplotypes in our diversity panel than in the European pooled varieties. In summary, the LD decay estimated in CIP germplasm was moderate, and comparable to the LD decay found in the European and North American potato germplasm (D’hoop *et al.* 2010; Vos *et al.* 2017; Sharma *et al.* 2018).

### QTL for late blight resistance

Several SNPs were significantly associated with late blight resistance in the field trials with a total of 16 markers tagging possible QTL (Tables 4 and 5). In the tetraploid data, 5 markers for late blight resistance were found in chromosomes 0, III, and IX, while in the diploid dataset 14 markers on chromosomes 0, III, V, VI, IX, and X were associated with the resistance phenotype. Populations of *P. infestans* are diverse and there is a trend of increasing diversity in potato-growing regions worldwide (Cooke and Lees 2004). Taking this into account, the markers on chromosomes IX and X could indicate a QTL for broad resistance not specific to regional late blight strains, while markers associated to the QTL on chromosomes III and V could be environment-specific (Tables 4 and 5). The highest number of SNPs associated to late blight resistance in the GWAS were mapped between 59 and 61.2 Mbp of chromosome IX in a region that had been previously associated with late blight resistance in Peru (Lindqvist-Kreuze *et al.* 2014). In addition, the markers were within or surrounding the segment that forms a large cluster of putative resistance genes. For example, the locus PGSC0003DMG400020587 encodes a homolog of *Rpi-vnt1* (Mosquera *et al.* 2016), a major gene for resistance to *P. infestans* that has been previously cloned and is characterized in the wild potato species *Solanum venturii* (Foster *et al.* 2009; Pel *et al.* 2009).

**Table 5:** GBS markers that were significantly associated with the late blight resistance phenotype in the field trials in China (Kun2015, Kun2016), Ethiopia (Hol2017), and Peru (Oxa2014)

Marker (chromosome followed by position)	Ref	Alt	Trial	Model	Threshold	Score	Effect
0_36073482	G	A	Oxa2014	Diplo-general	6	10.66	NA
				Diplo-additive	6.03	10.66	−0.20
				1-dom-alt	6.02	10.66	−0.20
			Kun2015	Diplo-general	6.01	25.19	NA
				Diplo-additive	6.03	25.19	−0.31
				1-dom-alt	6.02	25.19	−0.31
			Kun2016	Diplo-general	6.02	29.27	NA
				Diplo-additive	6.03	29.27	−0.29
				1-dom-alt	6.02	29.27	−0.29
3_45458723	A	C	Hol2017	Diplo-general	5.94	6.22	NA
				Diplo-additive	6.02	6.22	0.47
				1-dom-alt	6.02	6.22	0.47
3_45458753	G	A	Hol2017	Diplo-additive	6.02	6.22	0.47
				1-dom-alt	6.02	6.22	0.47
3_45458754	A	T	Hol2017	Diplo-additive	6.02	6.22	0.47
				1-dom-alt	6.02	6.22	0.47
5_5572873	G	A	Oxa2014	Diplo-additive	6.03	6.87	0.16
				1-dom-alt	6.02	6.87	0.16
6_45694949	G	A	Kun2015	Diplo-general	6.01	7.09	NA
				1-dom-alt	6.02	7.64	−0.15
			Kun2016	Diplo-general	6.02	7.33	NA
				1-dom-alt	6.02	8.13	−0.13
9_58779951	G	A	Oxa2014	Diplo-general	6	8.61	NA
				Diplo-additive	6.03	8.61	−0.17
				1-dom-alt	6.02	8.61	−0.17
			Kun2015	diplo-general	6.01	7.78	NA
				diplo-additive	6.03	7.78	−0.17
				1-dom-alt	6.02	7.78	−0.17
			Kun2016	Diplo-general	6.02	8.05	NA
				Diplo-additive	6.03	8.05	−0.16
				1-dom-alt	6.02	8.05	−0.16
9_59967523	A	T	Oxa2014	Diplo-general	6	6.01	NA
			Kun2015	Diplo-general	6.01	13.09	NA
				Diplo-additive	6.03	13.09	−0.21
				1-dom-alt	6.02	13.09	−0.21
			Kun2016	Diplo-general	6.02	16.79	NA
				Diplo-additive	6.03	16.79	−0.21
				1-dom-alt	6.02	16.79	−0.21
9_59997331	T	C	Oxa2014	Diplo-general	6	8.84	NA
				Diplo-additive	6.03	8.84	−0.17
				1-dom-alt	6.02	8.84	−0.17
			Kun2015	Diplo-general	6.01	18.25	NA
				Diplo-additive	6.03	18.25	−0.25
				1-dom-alt	6.02	18.25	−0.25
			Kun2016	Diplo-general	6.02	19.58	NA
				Diplo-additive	6.03	19.58	−0.23
				1-dom-alt	6.02	19.58	−0.23
9_60067335	A	G	Oxa2014	Diplo-general	6	15.15	NA
				Diplo-additive	6.03	15.15	−0.23
				1-dom-alt	6.02	15.15	−0.23
			Kun2015	Diplo-general	6.01	25.64	NA
				Diplo-additive	6.03	25.64	−0.31
				1-dom-alt	6.02	25.64	−0.31
			Kun2016	Diplo-general	6.02	27.56	NA
				Diplo-additive	6.03	27.56	−0.28
				1-dom-alt	6.02	27.56	−0.28
9_61106174	C	T	Kun2015	Diplo-general	6.01	6.89	NA
				Diplo-additive	6.03	7.54	−0.14
				1-dom-alt	6.02	7.05	−0.14
			Kun2016	Diplo-general	6.02	11.4	NA
				Diplo-additive	6.03	12.31	−0.15
				1-dom-alt	6.02	11.83	−0.15
9_61108928	T	G	Oxa2014	Diplo-additive	6.03	6.71	−0.12
				1-dom-alt	6.02	6.66	−0.12
			Kun2015	Diplo-general	6.01	8.94	NA
				Diplo-additive	6.03	9.87	−0.16
				1-dom-alt	6.02	9.56	−0.17
			Kun2016	Diplo-general	6.02	10.73	NA
				Diplo-additive	6.03	11.69	−0.15
				1-dom-alt	6.02	11.38	−0.15
9_61261167	A	C	Oxa2014	Diplo-general	6	14.23	NA
				Diplo-additive	6.03	13.88	−0.21
				1-dom-alt	6.02	15.15	−0.23
			Kun2015	Diplo-general	6.01	24.53	NA
				Diplo-additive	6.03	24.33	−0.30
				1-dom-alt	6.02	25.64	−0.31
			Kun2016	Diplo-general	6.02	26.41	NA
				Diplo-additive	6.03	26.33	−0.27
				1-dom-alt	6.02	27.56	−0.28
10_51544544	A	G	Oxa2014	Diplo-general	6	13.39	NA
				Diplo-additive	6.03	13.39	−0.22
				1-dom-alt	6.02	13.39	−0.22
			Kun2015	Diplo-general	6.01	23.14	NA
				Diplo-additive	6.03	23.14	−0.30
				1-dom-alt	6.02	23.14	−0.30
			Kun2016	Diplo-general	6.02	29.56	NA
				Diplo-additive	6.03	29.56	−0.29
				1-dom-alt	6.02	29.56	−0.29

The associations were modeled using diploid SNP with GWASpoly software and the diplo-general, diplo-additive, and 1-dominance models.

The 48x multiplexing of samples during the sequencing and stringent filtering for minimum read depth in all samples yielded relatively few SNP (less than 4 K). Therefore, to increase the chances of finding significant associations, the GWA was done using both tetraploid and the diplodized data. Three markers (9_58779951, 9_59967523, 9_60067335) map in the same region of chromosome IX previously found associated with late blight resistance in Peru (Li *et al.* 2010; Lindqvist-Kreuze *et al.* 2014). These markers are physically separated in the DM genome by 1.3 Mb, which fits the estimate for the LD decay in our diversity panel, suggesting that this could be a single QTL. The *R8* gene from *S. demissum* was recently identified within the QTL dPI09c reported by Li *et al.* (2010; Jiang *et al.* 2018). The QTL dPI09c interval begins at 60615044 bp in potato DM1-3 516 R44 (Potato Genome Sequencing Consortium 2011), which is over 0.6Mbp from the nearest GBS marker (9_60067335) and identified in the current research in the tetraploid marker set. None of the SNP in our tetraploid GBS marker set is located in the dPI09c interval (Jiang *et al.* 2018), and hence the markers in the tetraploid set could be indicative of another QTL. In the diploid marker set, the QTL in chromosome IX extends beyond that discovered in the tetraploid marker set, reaching also to the QTL dPI09c interval.

### Best linear unbiased models to predict late blight resistance

Phenotypic evaluation of potato germplasm in multiple environments is constrained by the difficulty of sharing vegetatively propagated tuber seed. Distribution of clonal material is restricted due to phytosanitary risks and strictly regulated. Furthermore, clonal seed multiplication rates are low from one generation to the next, and tuber seed is bulky and perishable. Therefore, it would be advantageous to be able to predict the performance of potato clones in a given environment based on their performance in another environment. This may be feasible for traits that exhibit low levels of genotype by environment interaction. Late blight resistance in CIP germplasm has been shown largely to be controlled by a large effect QTL (Li *et al.* 2012) and the late blight resistance gene *R8* has been identified in the QTL (Jiang *et al.* 2018). *R8* confers a broad-spectrum resistance with predicted global effect because virulence toward *R8* is only rarely encountered (Swiezynski *et al*. 2000; Haynes *et al.* 2002; Zhang and Kim 2007; Vossen *et al.* 2016, Lindqvist-Kreuze *et al.* 2020).

**Figure 3:**
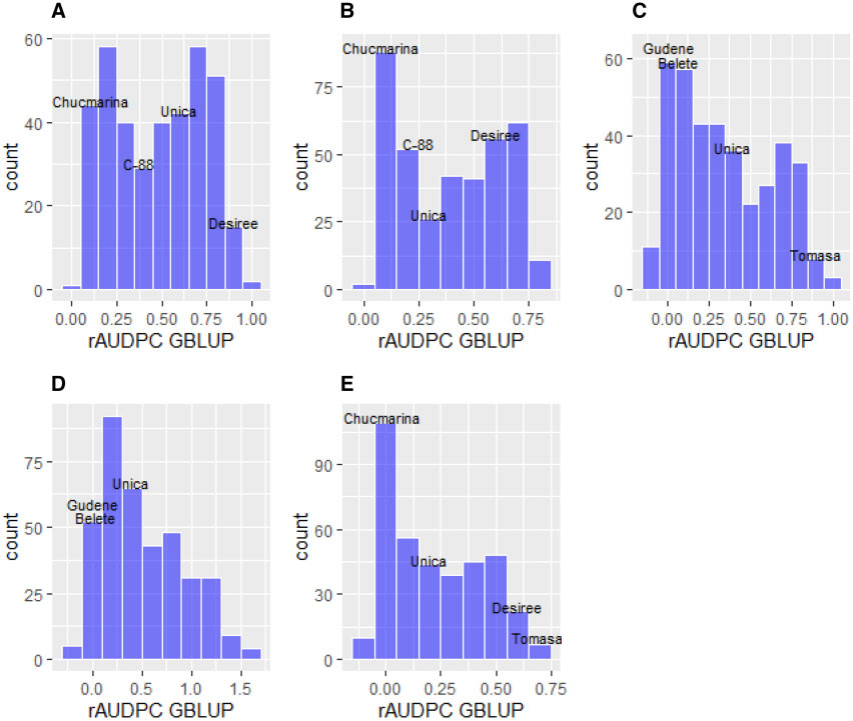
Histograms of genetic rAUDPC GBLUP values (diploid model with QTLxE fixed effects) in Kunming, China in 2015(A) and in 2016 (B), Holeta, Ethiopia in 2016 (C) and 2017 (D), and Oxapampa, Peru at 2014 (E). The shared control genotypes (checks) or well-known varieties in each trial are indicated in the plots based on their adjusted phenotypic rAUDPC value.

In this research project, more than 300 advanced tetraploid clones from CIP were shared with partners, but for various reasons not all were evaluated in all environments and the total overlapping set of genotypes that were evaluated in all environments was less than 60 (Table 2). Varieties with known levels of late blight resistance were included as controls in each environment and the high level of infection in the susceptible control varieties suggests that the environmental conditions were conducive for the disease development (Figure 3). The heritability of the late blight resistance phenotype was high in each trial (Table 2), indicating that the trait is most likely controlled by a major disease resistance gene or a QTL with a large effect. We set out to test the prediction ability of six different mixed models to estimate the genetic correlations across environments and to predict the late blight resistance of all clones in all environments. Relatively high prediction ability ranging from 0.815 to 0.864 was obtained with all the tested models (Table 6). The prediction ability of the mixed model improved after incorporating pedigree and marker information. Indeed, the fivefold cross-validation results demonstrated that both the pedigree PBLUP model and the diploid marker GBLUP model have superior performance compared to the BLUP model (Table 6). The highest prediction ability was obtained for the GBLUP model that utilized the diplodized marker set and included QTL(xE) fixed effects. The top 10 ranked genotypes in each environment, according to this model, are listed in Table 7. However, if molecular marker information is not available, a significant improvement for the prediction ability can be obtained from the pedigree information in the model. Another advantage of the PBLUP model is that predictions can also be obtained for the pedigree parents that were not phenotyped or genotyped (Supplementary Table S3). In summary, our results show that genomic prediction for late blight resistance in potato is feasible and has added value, confirming the results from previous studies (Stich and Van Inghelandt 2018; Enciso-Rodriguez *et al.* 2018). The complete prediction results with the best-performing models are listed in Supplementary Table S4.

**Table 6:** Estimated prediction ability of the different models for genetic and environment-specific predictions

Model	Prediction ability
BLUP	0.815 ± 0.009
PBLUP	0.855 ± 0.006
GBLUP (2x)	0.858 ± 0.006
GBLUP (4x)	0.855 ± 0.007
GBLUP (2x) with fixed QTL(xE) effects	0.868 ± 0.005
GBLUP (4x) with fixed QTL(xE) effects	0.864 ± 0.006

Fivefold cross-validation was obtained for the first two models.

**Table 7:** List of the ten most late blight resistant genotypes per environment based on the diploid GBLUP model with fixed QTL(xE) effects

Rank	Hol2016	Hol2017	Kun2015	Kun2016	Oxa2014
1	CIP396012.266	CIP398098.205	CIP301029.18	CIP395017.242	CIP398098.205
2	CIP393280.57	CIP398098.231	CIP399085.30	CIP396240.23	CIP398190.615
3	CIP396018.241	CIP398190.200	CIP393371.164	CIP395017.229	CIP396012.266
4	CIP301024.14	CIP396033.102	CIP399085.23	CIP393280.82	CIP398098.231
5	CIP396037.215	CIP393084.31	CIP395123.6	CIP396012.266	CIP394898.13
6	CIP392637.10	CIP398098.119	CIP395017.242	CIP393227.66	CIP384321.3
7	CIP396004.263	CIP393073.179	CIP399085.17	CIP301029.18	CIP398098.119
8	CIP384321.3	CIP393077.159	CIP384321.3	CIP384321.3	CIP301024.14
9	CIP393248.55	CIP398190.735	CIP391919.3	CIP392650.12	CIP398180.612
10	CIP304347.6	CIP398190.605	CIP399075.32	CIP395123.6	CIP398098.570

The CIP potato germplasm evaluated included many highly resistant genotypes based on the predicted rAUDPC values (GBLUPs) (Figure 3). The resistance of these genotypes is comparable to the resistant control genotype, which is released as a variety called “Chucmarina” in Peru and as “Belete” in Ethiopia (Figure 3). Notably, most of the genotypes tested in China were more resistant than the local variety “Cooperation-88” which has been popular for many years due to its good late blight resistance.

**Figure 4:**
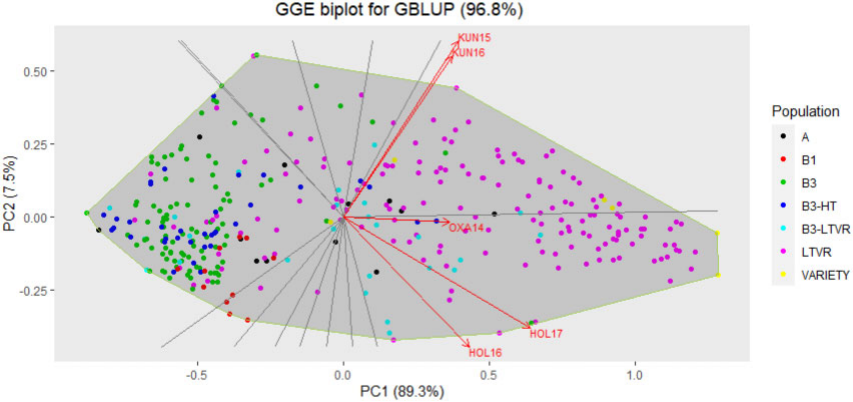
GGE biplot for predicted late blight resistance [based on the diploid GBLUP model with fixed QTL(xE) effects] of the potato genotypes in China, Kunming 2015 (KUN15) and 2016 (KUN16); Peru, Oxapampa 2014 (OXA14), and Ethiopia, Holetta 2016 (HOL16) and 2017 (HOL17).

**Figure 5:**
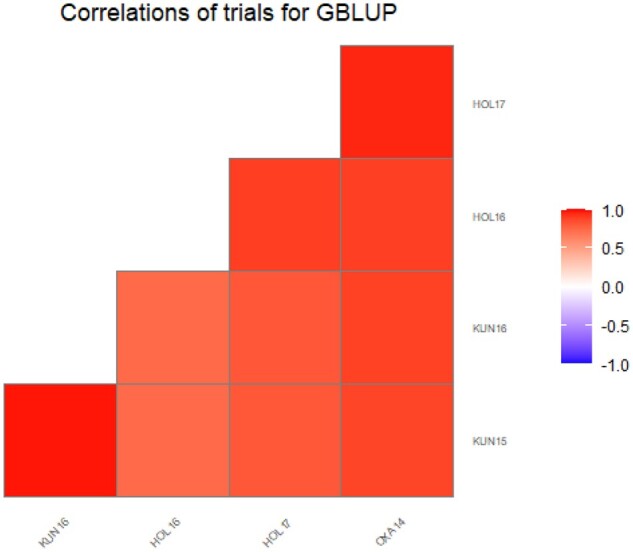
Heat-plot on the GBLUP correlations among the field trials in China (KUN15, KUN16), Ethiopia (HOL16, HOL17), and Peru (OXA14) based on the diploid marker GBLUP model with fixed QTL(xE) effects.

A biplot for predicted GBLUP values using the diploid marker-based kinship matrix shows the performance of the genotyped clones in all environments (Figure 4). In this figure, the genotypes that locate on the left side quadrats of the graph, and further away from the plot center, display the highest level of resistance. Genotypes near the horizontal mid-line on the left quadrats display stable resistance across environments, while genotypes toward the top and bottom extremes on the left quadrats display environment-specific resistance. Thus, some genotypes’ resistance to late blight is environment-specific, however, several genotypes show stable resistance across environments. The most resistant genotypes belong to the B3 and LB-HT populations, while only a few from the LTVR and B3-LTVR populations display high levels of resistance (Figure 4). The correlations among environments were high (Figure 5 and Table 8). The environment in Peru, especially, correlates highly with all the other environments, suggesting that resistant clones selected in Peru will also likely have good resistance in these other environments.

**Table 8:** Estimated genetic correlations between the five environments obtained by fitting the diploid marker GBLUP model

	Hol2016	Hol2017	Kun2015	Kun2016	Oxa2014
Hol2016	1.00	0.67	0.52	0.51	0.80
Hol2017	0.67	1.00	0.53	0.51	0.82
Kun2015	0.52	0.53	1.00	0.94	0.76
Kun2016	0.51	0.51	0.94	1.00	0.76
Oxa2014	0.80	0.82	0.76	0.76	1.00

In the 1990s, CIP’s breeding strategy focused on improving the quantitative resistance in the “B3” population by phenotypic recurrent selection under endemic pressure from the *P. infestans* in the Peruvian Andes, supplemented by progeny tests to identify parents with good general combining ability and to eliminate those resulting in segregation for hypersensitive response against test isolates. The pathogen population in this area is currently dominated by the A1 mating type and EC-1 clonal lineage, which is highly aggressive and complex in its virulence (Lindqvist-Kreuze *et al.* 2020). Despite differences in the mating type and clonal lineages of the pathogen populations among the countries, it seems that phenotypic selection for late blight resistance in Peru was largely successful and the results are transferable across the environments tested here.

## Conclusions

METs for foliar late blight resistance with a set of advanced tetraploid potato clones bred at International Potato Center in Peru identified QTL that were shared among environments and those that were environment-specific. QTL with the largest effect was identified in chromosome IX. Good predictive ability for the foliar LB resistance across environments was obtained using the BLUP model, and this was further improved by incorporating the pedigree and genotypic information in the mixed model. The best predictive ability was obtained with the GBLUP model for the diploid marker set using fixed QTL(xE) effects. The results show that many of the advanced clones bred in Peru for high levels of late blight resistance maintain their resistance in Ethiopia and China, suggesting that the centralized selection strategy has been largely successful.

## Data Availability

The field books containing the phenotypic data used in the analysis are available in the CIP Dataverse (https://data.cipotato.org/dataset.xhtml?persistentId=doi:10.21223/P3/JJJQV0) and (https://data.cipotato.org/dataset.xhtml?persistentId=doi:10.21223/6TRC9T). Population denominations and parentage of the potato genotypes are detailed in Supplementary Table S1. The genotypic best linear unbiased estimators (BLUEs) of rAUDPC for each environment are in Supplementary Table S2; the pedigree best linear unbiased predictors (PBLUPs) are in Supplementary Table S3; and the genomic best linear unbiased predictors (GBLUPs) using the model with the best prediction ability are in Supplementary Table S4. The genotypic data are available in variant calling format (vcf), in diploid format (10.6084/m9.figshare.12786398), and in tetraploid format (10.6084/m9.figshare.12789383).
